# Animal movement estimation and network-based epidemic modeling: Illustration for the swine industry in Iowa (US)

**DOI:** 10.1371/journal.pone.0326234

**Published:** 2025-06-18

**Authors:** Qihui Yang, Beatriz Martínez-López, Sifat Afroj Moon, Jose Pablo Gomez-Vazquez, Caterina Scoglio

**Affiliations:** 1 Department of Electrical and Computer Engineering, Kansas State University, Manhattan, Kansas, United States of America; 2 Center for Animal Disease Modeling and Surveillance (CADMS), Department of Medicine & Epidemiology, School of Veterinary Medicine, University of California, Davis, California, United States of America; 3 Computing and Computational Sciences Directorate, Oak Ridge National Laboratory, Knoxville, Tennessee, United States of America; Universidad Santo Tomas, CHILE

## Abstract

Animal movement plays a critical role in disease transmission between farms. However, in the United States, the lack of available animal shipment data, sometimes coupled with a lack of detailed information about farm demographics and characteristics, presents great challenges for epidemic modeling and prediction. In this study, we proposed a new method based on the maximum entropy to generate “synthetic” animal movement networks, considering available statistics about the premises operation type, operation size, and the distance between premises. We illustrated our method for the swine movement networks in Iowa and performed network analyses to gain insights into the swine industry. We then applied the generated networks to a network-based epidemic model to identify potential system vulnerabilities in terms of disease transmission. The model was parameterized for African Swine Fever (ASF) as the US swine industry is quite concerned about this disease. Results show that premises with a central role in the network are more vulnerable to disease outbreaks and play an important role in disease spread. Simulations with outbreaks starting from random farms reveal no significant large outbreaks, indicating the system’s relative robustness against arbitrary disease introductions. However, outbreaks originating from high out-degree farms can lead to large epidemic sizes. This underscores the importance for stakeholders and policymakers to continue improving animal movement records and traceability programs in the US and the value of making that data available to epidemiologists and modelers to better understand risk and inform strategies aimed to cost-effectively prevent and control disease transmission. Our approach could be easily adapted to estimate movement networks in other animal production systems and to inform disease spread models for various infectious diseases.

## 1. Introduction

The swine industry in Iowa holds significant economic importance as the largest in the United States, contributing approximately $9.40 billion in annual cash receipts from hog sales [1]. However, potential disease outbreaks pose threats to the industry, leading to devastating economic consequences. Endemic diseases such as porcine reproductive and respiratory syndrome virus are responsible of more than $664 million annual production losses to the US swine industry [2]. A more recent work estimated that the potential introduction and spread of African swine fever (ASF) would cause $1.78 billion in economic losses and 22,076 job losses in Iowa over 10 years [3]. Such staggering costs have prompted urgent efforts to improve swine industry diagnostic capacity, enhance traceability and develop epidemic models to rapidly detect and mitigate future disease outbreaks.

It is well known that disease spread through animal movements, i.e., direct contact, are often identified as the primary driver of disease transmission [4], compared to indirect contacts between animals and wildlife, as well as contacts through contaminated feed or fomite [5,6]. Therefore, a good understanding of animal movement patterns is crucial for developing accurate epidemic models and informing outbreak preparedness plans [7]. The availability of livestock shipment databases has significantly contributed to understanding disease spread patterns in European countries [8,9], Australia [10]. However, no mandatory database for livestock shipments currently exists in the US, mainly due to privacy concerns [11]. The US shipment data mainly come from private sources provided by large swine companies or interstate data, such as certificates of veterinary inspection (CVIs) data. The former usually includes movement data among premises owned by certain large companies [12,13]. The latter does not capture within-state movement and is collected during mandatory veterinary inspections when animals are transported across state borders.

In response to the lack of comprehensive swine movement data, several studies have attempted to generate swine movement networks in the US. Valdes-Donoso et al. utilized random forest algorithms and confidential survey data from two counties to predict swine movements in the 34 counties of Minnesota [14]. Moon et al. estimated movement probabilities among subpopulations of different size groups for the state of Iowa, based on the maximum information entropy approach using published county-level sales and inventory data from the United States Department of Agriculture National Agricultural Statistics Service (USDA-NASS) [15]. Similarly, a recent study used a Bayesian statistical model based on US interstate swine shipment data to predict premises-level swine movements for the whole US [16]. The study has limitations in capturing within-state shipments, as their networks are predominantly inferred from interstate shipment data, assuming that the driving factors behind within-state and between-state shipments are the same. Furthermore, most of these works did not consider the premises operation type, which is of much interest to veterinarians. Moreover, given that the US swine production industry is heavily vertically integrated, premises operation types directly determine the movement patterns. For instance, pigs may move from nurseries to finishers, but not in the reverse direction. While Ferdousi et al. developed a network generator that considers assortative mixing between different combinations of operation types reported in [14], they did not incorporate the effects of operation sizes and distances between premises [17]. Additionally, due to a lack of farm demographic data, previous studies either allocated pigs randomly to farms based on the swine premises size distribution from the NASS data, or utilized synthetic pig population data generated by Burdett et al. [18].

In addition to the studies on animal movement networks, researchers have made modeling efforts to estimate ASF epidemiological parameters and provide risk assessment of disease introduction and/or spread [19–22]. Lee et al. [20] used a network-based model at the farm level to simulate ASF spreading within a hypothetical population of 1,000 pig farms in Vietnam. Muñoz et al. [21] applied a probabilistic approach to assess the ASF risk introduction into Spain. The model is a decision-making tool for prioritizing preventive measures against disease entry, and includes multiple introduction pathways such as the import of pork products and contacts with wild boar. Many models are based on the classic susceptible-exposed-infectious-removed structure, and assume a homogeneous and random mixing population for the within-farm structure. While some studies have attempted to model ASF outbreaks in the US [19], few of them have used spatial-explicit models that account specifically for the contact patterns between farms. Sykes et al. [19] developed a farm-level spatially-explicit stochastic compartmental model to simulate ASF spreading using real movement data from three swine production companies in the southeastern US.

In this paper, we generate synthetic farm-level movement networks specifically for the state of Iowa, and conduct network analyses. The networks are generated using publicly available swine premises data based on the maximum entropy approach from [15,23]. In the network, nodes represent swine premises, and directed weighted links reflect the number of pigs transported between premises. We also develop an individual-level epidemic model to provide spatial relative risk distributions under various scenarios, where nodes represent pigs and links incorporate information from the farm-level networks. Our results demonstrate the value of network centrality measures such as out strength centrality in identifying critical farms. No previous study has simultaneously considered the effects of premises operation type, operation sizes and distances between premises, when generating synthetic movement networks in Iowa. The major contribution of this work lies in our inclusion of premises operation types when generating synthetic networks, as later demonstrated, different operation types can greatly impact the network characteristics. Including considerations of operation types during the network generation with an individual-level epidemic model, this study fills a key gap in existing literature and provides a versatile framework for risk assessment in data-limited settings.

## 2. Materials and methods

In this section, we introduce the movement network generation and the epidemic model for risk assessment.

### 2.1 Movement network estimation

We generate farm-level animal movement networks, where nodes represent farms and edges indicate the number of animals moved between farms.

#### 2.1.1 Study population and data sources.

This study utilized the *premises demography data* from the Animal Feeding Operation (AFO) database maintained by the Iowa Department of Natural Resources [24]. The database contains data from 7,607 farms located across 99 counties in Iowa. In total, there are 26,644,194 pigs. The dataset contains essential information such as premises identification number, animal headcounts for five different animal types (swine gestation & boars, swine gilts & isolation, swine grow to finish, swine nursery, swine sow and litter, and swine wean to finish), as well as the location coordinates. Based on the animal type information, we categorize farms into the five operation types: finishing farm, gilt development unit (GDU), nursery, sow farm, and wean-to-finish farm. The *operation type mixing matrix*, representing the fraction of pig movements from one operation type (origin farm) to another (destination farm) relative to the total pig movements, was obtained based on the number pigs transported from [12] and is detailed in Table S1 in supplemental material.

#### 2.1.2 Movement estimation based on maximum entropy.

For network generation, we distribute animal movement flows based on different distance categories and farm sizes of premises. More specifically, we collected data including swine inventory, sales, slaughter, and dead/lost census data from the USDA-NASS for 2017. Following the maximum entropy approach proposed by [15,23], we first estimated non-disclosed data points in the inventory and sales data, and then estimated movement probabilities among different sub-populations considering several linear constraints. One subpopulation (*c*, *s*) is denoted as the swine population in a size group *s* in a county *c*, where *c* = 1, 2, …, *C* and *s* = 1, 2, …, *S*. *C* is the number of counties in the system, and *S* is the number of size groups. The objective of the estimation problems, as shown below, aims at maximizing the entropy with minimum assumptions.


$$\begin{array}{cc} \max\left\{Entropy\right\} & =max\{-\sum_{c_{1}\in C}\sum_{s_{1}\in S}\sum_{c_{2}\in C}\sum_{s_{2}\in S}m_{s_{1},s_{2},dist(c_{1},c_{2})}^{d}\times \log\left(m_{s_{1},s_{2},dist(c_{1},c_{2})}^{d}\right)\\ & \;-\sum_{c_{1}\in C}\sum_{s_{1}\in S}{os}_{c_{1},s_{1}}^{d}\times \log\left({os}_{c_{1},s_{1}}^{d}\right)-\sum_{c_{1}\in C}\sum_{s_{1}\in S}{rn}_{c_{1},s_{1}}^{d}\times \log\left({rn}_{c_{1},s_{1}}^{d}\right)\\ & \;-\sum_{c_{1}\in C}\sum_{s_{1}\in S}{sl}_{c_{1},s_{1}}^{d}\times \log\left({sl}_{c_{1},s_{1}}^{d}\right)-\sum_{c_{1}\in C}\sum_{s_{1}\in S}{lt}_{c_{1},s_{1}}^{d}\times \log\left({lt}_{c_{1},s_{1}}^{d}\right)\} \end{array}$$ (1)

Where $m_{s_{1},s_{2},dist(c_{1},\;c_{2})}^{d}$ represents the movement probability from subpopulations (*c*_1_, *s*_1_) and subpopulation (*c*_2_, *s*_2_). The index variables *c*_1_ and *s*_1_ are county index and size group index of the originating subpopulation. The index variables *c*_2_ and *s*_2_ are county index and size group index of the receiving subpopulation. ${os}_{c_{1},s_{1}}^{d}$ represents the movement probability from subpopulation (*c*_1_, *s*_1_) to the outside state in a week. ${rn}_{c_{1},s_{1}}^{d}$ is the probability to stay in subpopulation (*c*_1_, *s*_1_) in a week. ${sl}_{c_{1},s_{1}}^{d}$ refers to the probability of pigs in subpopulation (*c*_1_, *s*_1_) becoming slaughtered in a week. ${lt}_{c_{1},s_{1}}^{d}$ is the probability that pigs in subpopulation (*c*_1_, *s*_1_) becoming lost or dead in a week. The superscript *d* is the decision parameter.

In total, there are 99 counties for Iowa, and seven size groups, namely, 1–24 pigs, 25–49 pigs, 50–99 pigs, 100–199 pigs, 200–499 pigs, 500–999 pigs, and more than 1000 pigs. We select these size groups as USDA-NASS reports pig population at the county level only for these groups. The distances between pairs of counties (based on county centroids) are divided into five categories: (1) 0≤ distance <20km, (2) 20km≤ distance <100 km, (3) 100km≤ distance <200km (4) 200km≤ distance <400km (5) distance ≥ 400 km. The obtained movement probabilities among sub-populations with different distance categories and size groups are presented in Table S2 in the supplemental material. As we updated the input data from 2012 (as used in [15]) to 2017 and there are minimal differences between these datasets, the values in Table S2 closely match those reported in [15].

Using the above-mentioned input data, we generate farm-level networks with the following steps. More details for implementation can be seen in Supplemental material **Algorithm 1**.

1) Assign each farm node an operation type, location, and the number of pigs based on premises demography data.
2) Generate sub-populations (*c*, *s*, *t*), where *c* = 1, 2, 3, …., *C*; *s* = 1, 2, 3, … *S;* and operation type *t* ∈ {Boar stud, finishing farm, GDU, nursery, sow farm, wean-to-finish}.
  3) Calculate the number of pigs to be moved, denoted by *Q*(*t*_1_, *t*_2_) between operation types *t*_1_ and *t*_2_, based on the number of pigs moved each week within Iowa and the mixing matrix.
  4) For operation types *t*_1_ *and t*_2_, pigs *p*_1_ and *p*_2_ from sub-population (*c*_1_, *s*_1_, *t*_1_) and sub-population (*c*_2_, *s*_2_, *t*_2_) are randomly selected to get connected representing the pig shipment, based on movement probabilities (Table S2 in the supplemental material). Repeat the process until *Q*(*t*_1_, *t*_2_) pigs are transported between *t*_1_ and *t*_2_ (Line 23–47 in Algorithm 1 in supplemental material).
  5) If there is no link from the parent farm *f*_1_ of pig *p*_1_ to the parent farm *f*_2_ of pig *p*_2_, create a link from *f*_1_ to *f*_2_. Otherwise, increase its weight by 1.

We perform network analyses through the Python igraph package. For the generated networks, nodes represent premises, and edges are directed and weighted by the number of pigs moved. The following network properties are reported: in/out-strength centrality [25], betweenness centrality [25], eigenvector centrality [26], density [26], transitivity coefficient [27], diameter [27,28], strongly/weakly connected component [27]. In addition, we present the log-log strength distribution to check whether the networks exhibit the scale-free property. The Barabási-Albert [29] network model is known for its scale-free characteristics, where most nodes have low node degrees and a few nodes are featured with high node degrees.

### 2.2 The individual-level network-based epidemic model

We develop an individual-level network model for risk assessment based on the Generalized Epidemic Modeling Framework (GEMF) [30,31], adapting the GEMF toolbox in Matlab. The toolbox is capable of simulating infectious disease models with multiple network layers of interactions. GEMF simulates iterations of the stochastic Markov process corresponding to the epidemic model. Each simulation is event-based and stops either when a maximum number of events or the simulation time is reached. It is important to highlight that we just focused on modeling the disease spread during the high-risk period (i.e., HRP, = time from the initial infection to detection/notification of the disease and establishment of the mitigation and control measures), therefore we did not implement any intervention or control strategy. The HRP was assumed to be one month based on previous studies [21,32–35].

**Fig 1:**
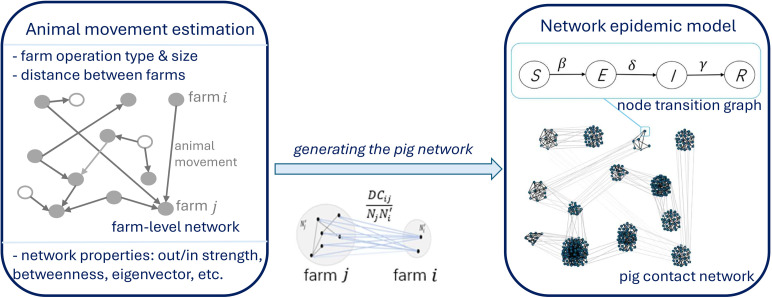
Schematic diagram of the model.


Fig 1 shows the schematic diagram of the model. As shown in Fig 1, we utilize a susceptible (S)-exposed (E)-infected (I)-removed (R) model. The time distributions of processes determining the infection events of a node are assumed to be exponentially distributed and statistically independent from each other. The node-level Markov process for node i = 1, 2,... *N*, is expressed as follows

$$P_{r}\left[x_{i}(t+\Delta t)=1|x_{i}(t)=0,\;X(t)\right]=\beta Y_{i}\Delta t+o(\Delta t)$$ (2)

$$P_{r}\left[x_{i}(t+\Delta t)=2|x_{i}(t)=1,X(t)\right]=\delta \Delta t+o(\Delta t)$$ (3)

$$P_{r}\left[X_{i}(t+\Delta t)=3|x_{i}(t)=2,X(t)\right]=\gamma \Delta t+o(\Delta t)$$ (4)

Where $x_{i}=$1, 2, 3 representing the state of node i being in the *S*, *E*, *I*, *R*, respectively. The value X(t) is the joint state of all nodes at time t, and $Y_{i}$ is the number of infected neighbors of node *i*.

We generate the adjacency list representing the network topology, where nodes represent pigs. Links between nodes reflect both pig-to-pig contacts within a farm and pig movements between farms, affecting the disease spread. The adjacency list is then imported to GEMF as the input network for the stochastic simulations. To reduce the computational cost, scaling factor is applied and the final network includes 466,316 nodes. Details of the scaling procedure and the generation of pig networks based on farm network information are detailed in the supplemental material.

Initially, all the nodes are susceptible, except for those from index farms are set as infected to start the epidemic. Infected node can transmit the disease to its susceptible neighbors in the pig contact network. A node changes its state through either nodal transitions or edge-based transitions. Nodal transitions of a node do not depend on the states of its neighbors in the network, while edge-based transitions of a node are caused by interactions between neighbors in the network. Since infection processes are statistically independent, the transition rate for a susceptible node to become exposed is determined by the transmission probability β and the number of infected neighbor nodes $Y_{i}$. An exposed node will become infected with a rate δ=1/4 day^-1^, which is the inverse of the mean latent period [36]. The node transitions from an infected state to a removed state at removal rate γ=1/7 day^-1^, leading to exponentially distributed infectious periods with mean equal to 7 days [8,36]. Transmission probability β=0.1  and β=0.05 are considered based on references [22,33].

Because the data used in this study were obtained from publicly available databases, and no experiments were performed on live animals, Institutional Animal Care and Use Committee approval was not required.

## 3. Results

In this section, we present results of the network analyses and stochastic simulations for the network-based model.

### 3.1 Synthetic swine movement works

In total, we generate 200 synthetic weekly networks realizations. Given the relative stability of the swine industry in the US throughout the year [12], we did not consider seasonality and these networks are static networks. For each network, we calculate various network properties such as average shortest path and average node degree. These properties are then aggregated across all networks to obtain mean values along with the corresponding minimum and maximum values, which are presented in Table 1. To compare network properties from other sources, we aggregate four weekly networks to approximate a monthly network, resulting 50 monthly networks. Repeated edges with the same pair of source origin and destination are aggregated into the same edge with summation of the edge weights.

**Table 1:** General topological properties of farm-level swine networks from this study and two prior studies. The first column lists the metrics and the subsequent columns show the average (minimum, maximum) values for each study.

	This paper (1 week)	This paper (1 month)	Passafaro et al. [13]	Lee et al. [12]
Scope of research	Data from state of Iowa AFO database, the US	Data from state of Iowa AFO database, the US	One multi-site system in Iowa, the US	One multi-site system in one state, the US
Time scale	1 week	1 month	1 month	1 month
Number of edges	36955 (36472, 36981)	83370 (82788, 83750)	358 (247, 432)	381 (270, 484)
Number of nodes	7406 (7375, 7438)	7603 (7597, 7606)	267 (225, 306)	249 (219, 280)
Density ($\times 10^{4}$)	6.74 (6.63, 6.79)	14.42 (14.31, 14.49)	9.00 (6.00, 11.00)	62.00 (46.00, 79.00)
Diameter	17.44 (13.00, 25.00)	9.78 (9.00, 11.00)	7.00 (4.00, 11.00)	9.00 (5.00,13.00)
Average shortest path	3.29 (3.15, 3.52)	2.91 (2.89, 2.93)	2.50 (1.50, 3.30)	3.20 (1.90, 5.98)
Transitivity (undirected)	0.036 (0.035, 0.037)	0.035 (0.034, 0.036)	0.014 (0.001, 0.043)	0.027 (0.002, 0.054)
Number of weakly connected components	4 (1, 10)	1 (1, 1)	N/A	8 (2, 17)
Number of strongly connected components	4116 (3956, 4266)	1778 (1718, 1823)	N/A	226 (192, 258)
Size of the largest strongly connected component (%)	1.09 (0.93, 1.29)	2.86 (2.59, 3.14)	0.45 (0.00, 0.97)	0.03 (0.01, 0.17)
Size of the largest weakly connected component (%)	97.27 (96.87, 97.71)	99.95 (99.87, 99.99)	37.3 (23.9, 44.8)	93.2 (87, 99)
Mean betweenness	295.59 (269.00, 336.96)	520.54 (508.24, 534.51)	N/A	N/A
Mean eigenvector ($\times 10^{4}$)	3.80 (1.35, 8.46)	3.66 (1.31, 6.67)	N/A	N/A
Average in-degree	4.99 (4.92, 5.01)	10.97 (10.88, 11.02)	N/A	N/A
Average out-degree	4.99 (4.92, 5.01)	10.97 (10.88, 11.02)	N/A	N/A

**Fig 2:**
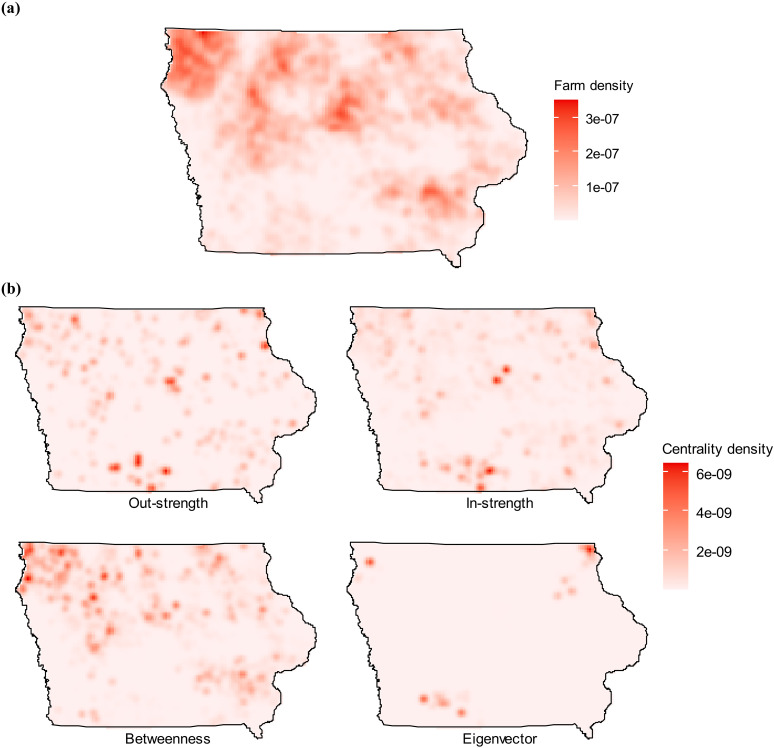
Distributions of (a) farm density and (b) network centrality measures: out-strength, in-strength, betweenness, and eigenvector centrality of the swine movements at the level of premises. On the map, the raster with the red color scale was produced using the median value of the network metrics from the 200 synthetic networks.

Fig 2 shows the spatial distribution of the swine farms in Iowa as well as the out-strength, in-strength, betweenness, and eigenvector centralities of the generated weekly networks. The map indicates that most premises show low values for centrality measures, implying they transport few or no animals. Some areas are highlighted with high centrality measures. For instance, the bottom middle area exhibits significantly higher out-strength centralities than the rest of the region.

**Fig 3:**
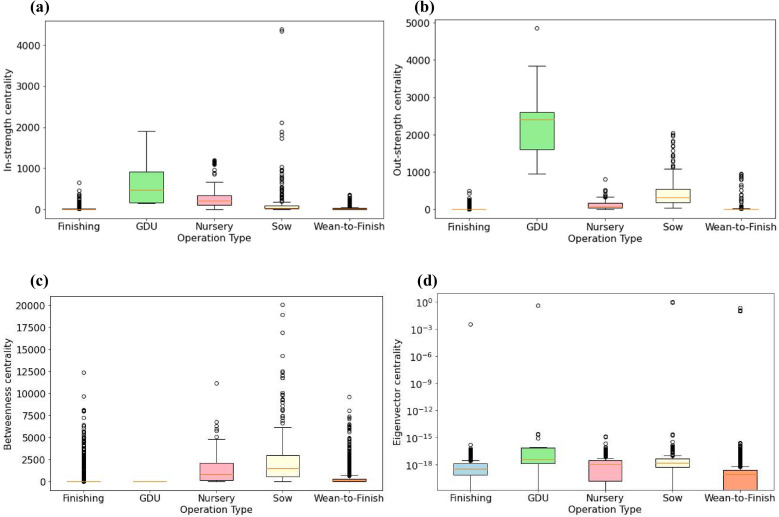
Boxplots representing the distribution of the centrality measures for each production type: (a) in-strength, (b) out-strength, (c) betweenness and, (d) eigenvector.

Fig 3 presents the distributions of centrality measures categorized by production type. Overall, GDUs and nursery farms show the largest and second largest in-strength centrality, respectively. Sow farms exhibit the third largest in-strength centralities with many outliers. Fig 3(b) shows that GDUs have the highest out-strength centrality, followed by sow farms. In Fig 3(c), sow farms have the greatest betweenness centrality, followed by nursery farms, indicating their importance in the network connectivity.

**Fig 4:**
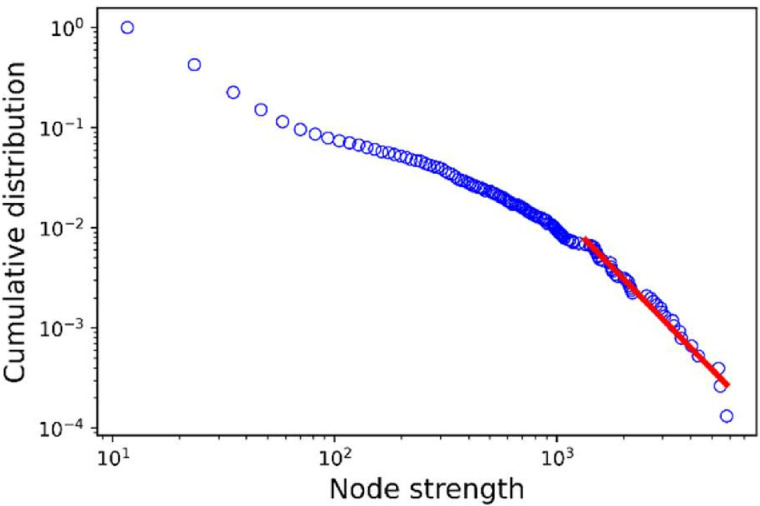
Log-log plot of the cumulative distribution of node strength. The blue circles indicate the node strength distribution while the red line is estimated by the linear regression.

Fig 4 shows the cumulative distribution of the node strength on a logarithmic scale. The figure, based one network realization, demonstrated that the network exhibits some scale-free property, with a few high-degree nodes and many low-degree nodes. In addition, there is an exponential decay in the tail as indicated by the straight line.

### 3.2 Outbreak dynamics

The network-based model is applied to the state of Iowa with parameters described in Section 2.2. In the following, we describe and visualize the simulation results with risk maps both at farm-level and county level.

#### 3.2.1 Selection of index farms randomly from the whole system.

**Fig 5:**
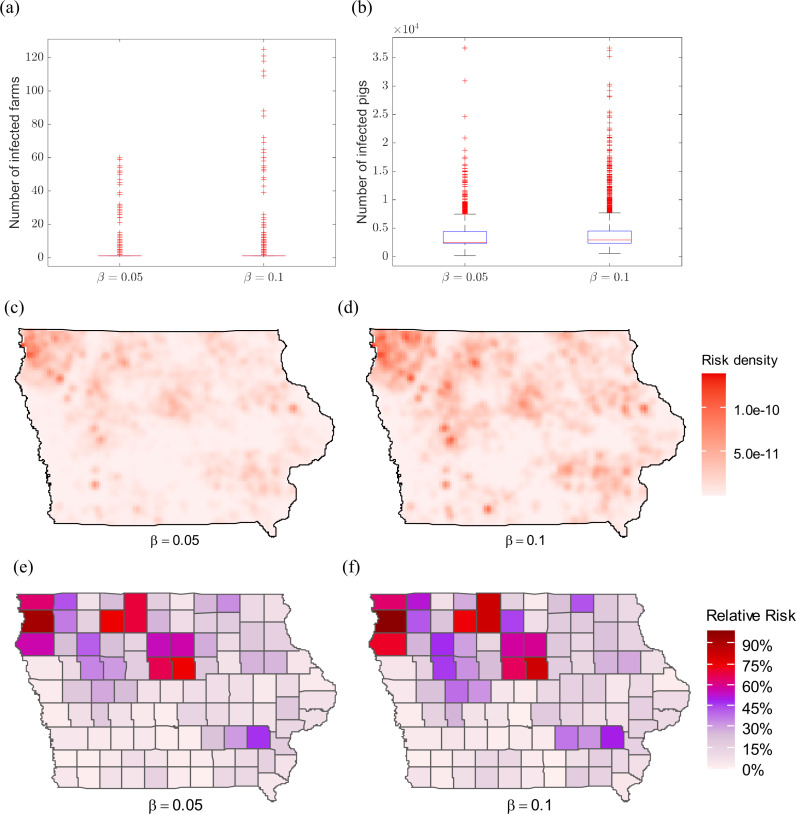
Simulation results of our ASF network-based model using a random selection of the index farm. Boxplots in (a) show the number of infected farms with transmission probability β=0.05 and β=0.1. Boxplots in (b) show the number of infected pigs with β=0.05 and β=0.1. Maps (c – d) depict ASF infection risks on the map of Iowa with β=0.05  and β=0.1,  respectively. The ASF risk of infection is calculated as the number of times that farm gets infected over all simulation runs. Maps (e – f) are relative county-level risk maps with β=0.05  and β=0.1. The relative risk for each county is computed based on the mean number of infected pigs over all simulation runs. One farm is selected as the index farm to start the epidemic. 5,000 simulation runs are performed.

Fig 5 shows the simulation results obtained using random selection of index farms to start the epidemics. A total of 5000 simulation runs are performed for each scenario. The simulation period is 31 days, which is the assumed maximum duration of the high-risk period. We observe that outbreaks starting at random farms lead to no-spreading outbreaks for most simulation runs. In Fig 5(a), the median number of infected farms is one for both scenarios β=0.05 and β=0.1. Fig 5(b) shows the number of infected pigs, with a maximum reaching 36,704. Fig 5(c–d) presents the farm-level ASF infection risk, calculated as the number of times that the farm gets infected over all simulation runs.

Fig 5(e–f) shows the relative county-level risks (i.e., normalized by setting the highest county risk to 1.0) with random selection of index farms. The risks are computed based on the mean number of infected pigs over all simulation runs. The results show that there is a large outbreak cluster in the northwestern area of the state of Iowa, which are high-density regions of pig farms.

#### 3.2.2 Selection of index farms based on centrality measures.

**Fig 6:**
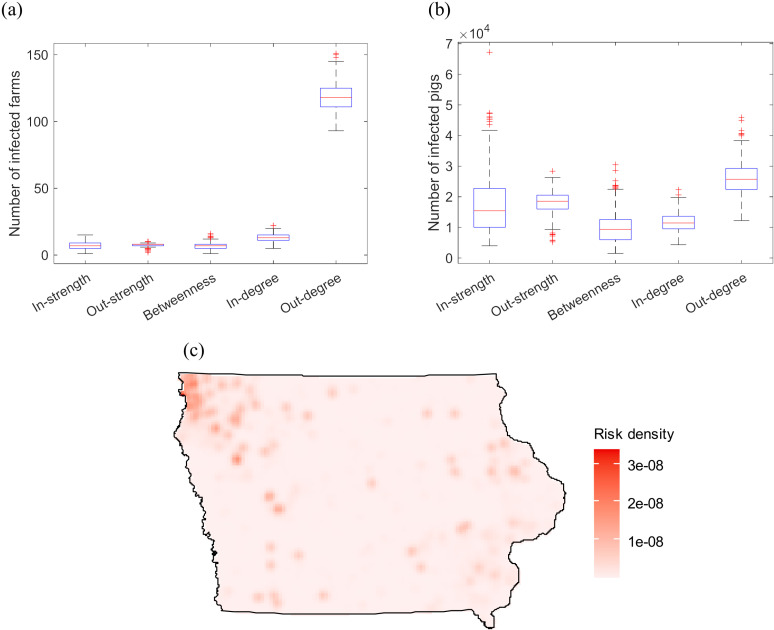
Simulation results with selection of index farms based on centrality measures and transmission probability β=0.1. Panel (a) shows the number of infected farms. Panel (b) shows the number of infected pigs. Panel (c) depicts the risk map for selecting index farms based on the out-degree centrality. One farm is selected as index farms to start the epidemic. 500 simulation runs are performed for each scenario.

Fig 6 depicts the simulation results obtained using centrality measure-based selection strategies. For each scenario, the farm with the highest value for a given metric is selected to start the epidemic. 500 runs are performed for each scenario. The selection strategy based on out-degree centrality shows the highest mean number of infected pigs, with 26,043 (95% CI: 25,581–26,506) pigs infected. Numbers of infected pigs under other scenarios (selecting index farms based on in-degree, in-strength, out-strength, betweenness centralities) range from 9,820 (95% CI: 9,383–10,257) to 18,073 (95% CI: 17,764–18,383).

#### 3.2.3 Selection of index farms based on operation types.

**Fig 7:**
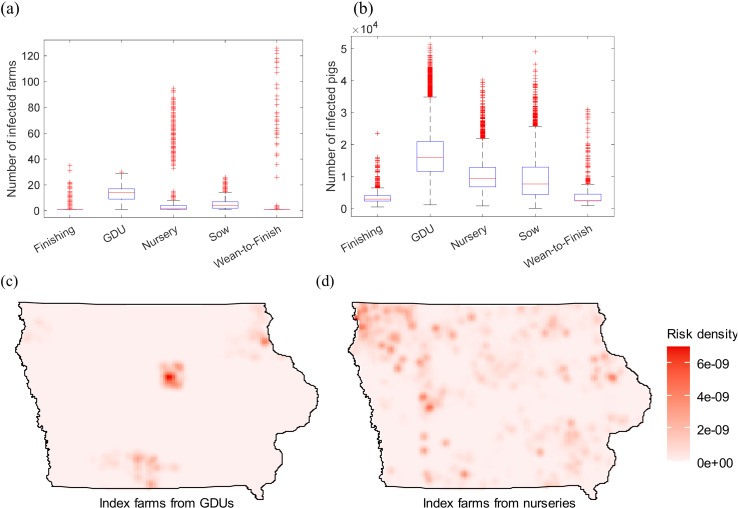
Simulation results with selection of index farms based on operation types and transmission probability β=0.1. Panel (a) shows the number of infected farms. Panel (b) shows the number of infected pigs. Panels (c) and (d) depict the risk maps for randomly selecting index farms from GDUs and nurseries, respectively. One farm is selected as index farms to start the epidemic. 5000 simulation runs are performed for each scenario.

Fig 7 illustrates the simulation results using operation type-based selection strategies. 5,000 runs are performed for each scenario, in which index farms are randomly selected from those with a specific operation type. Selecting from GDUs results in the highest infections, with a mean number of 17,209 (95% CI: 16,959–17,459) pigs affected. The other scenarios (selecting from finishing, nursery, sow, or wean-to-finish farms) show a mean number of 3,316 (95% CI: 3,271–3,361) to 10,370 (95% CI:10,212–10,528) pigs infected.

## 4. Discussion

In this study, we proposed a new method to generate synthetic swine movement networks, considering factors such as individual farm’s operation type, operation size, and distances between them, and pig-level contacts, which has been rarely considered in previous studies. We then conducted network analyses to gain insights into the network contact structures and use this information to run an individual-based network model to simulate the ASF transmission during the HRP. Based on this, we examined the magnitude of ASF epidemics under different epidemiological scenarios (i.e., selecting diverse index cases) and compare the outcomes.

The results from network analyses revealed distinct heterogeneities in the individual premises-level network properties (Fig 2), indicating that certain premises play a more central role in the system and are potentially more vulnerable to disease introduction and spread. The node-strength distribution of the networks exhibits scale-free properties with a fast exponential decay in the tail very similar to other papers describing cargo or passenger transportation networks [37]. GDUs exhibit the highest in-strength and out-strength centralities and display greater variability than other production types. Nursery and sow farms emerged as vital components of network connectivity, as evidenced by their higher betweenness values.

The network characterization enables investigation into the epidemic prediction and control, where animal movements have large effect on the epidemic spread. Our simulation results demonstrated that randomly selecting index farms to start the epidemics led to most simulation runs associated with no spreading outbreaks, indicating the system’s relative robustness to the arbitrary disease introductions. However, targeted selecting index farms based on centrality measures is more likely to lead to large outbreaks. Particularly, infecting farms with the highest out-degree leads to 26,043 pig infections on average (Fig 6).

Understanding the production system’s contact structures is crucial for realistically model epidemics of animal diseases and provide evidence-based science to support more cost-effective mitigation strategies. US is one of the few developed countries in the world with lack of livestock traceability. This is more surprising considering that US is a top exporter in many livestock products and any outbreak, even if localized in a certain state, could cause immediate disruption of those exports at national scale (as regionalization is difficult to implement without a proper tracing system). Although the swine industry is proactively working to change that, there is a need to accelerate the process not only in swine but in other livestock species given the global pressing pandemics such as African Swine fever, Avian influenza and the re-emergence of other livestock diseases such as bovine tuberculosis, brucellosis, lumpy skin disease, etc. The economic cost of stablishing a traceability system in the US is null compared to the benefits that brings to producers, consumers, and other stakeholders [38]. Many countries in Latin America, Europe and Asia, with less GDP than the US have been able to effectively establish an effective livestock traceability system. Therefore, the main issue is not really cost but the concerns about privacy issues. The establishment of a traceability system will facilitate the rapid detection and response of any endemic and emerging threat as well as will open new markets translating in hundreds of millions of dollars of benefits for livestock producers, similar to the experiences in Australia and Europe [38].

In this paper, we inferred movement patterns using the maximum-entropy approach from [15], which considers operation sizes and distance between premises. We assumed a constant value for the total number of swine moved within a given period and did not account for the potential impact of seasonality on animal movement. However, we believe that this should be a reasonable assumption as other authors suggested that shipments in the US swine industry are relatively constant and, therefore, there are not many differences between seasons [12].

Another limitation of this study is the use of synthetic movement networks. As aforementioned, the locations and swine inventories of farms are highly confidential, and the actual swine movement data within Iowa are not publicly available mainly due to privacy concerns. Accordingly, the accuracy of the outcomes cannot be directly validated using real data. This is why the first objective of this study was to generate synthetic networks. To achieve this goal, we inferred movement patterns within Iowa through a maximum entropy approach [15, 23] and generate networks incorporating farm operation types. Overall, the swine industry in Iowa is relatively stable [12]. To demonstrate that our network generation approach is reasonable, we obtained movement patterns (in Table S3 in supplemental material) with data set in [16]. This synthetic data set was generated based on inter-state shipment data, without considering farm types. Based on this alternative movement pattern, we include operation types and generate an additional set of movement networks using the approach proposed in this paper. We perform network analyses and the results are shown in Fig S1 and Fig S2 (in supplemental material). Comparing Fig S1(b) and Fig 2(b), we observe similar trends in areas with higher node centrality measures. For example, the southern bottom part of the map has nodes with greater out-strength in both figures. In addition, Fig S2 and Fig 3 exhibit similar distributions of centrality measures in terms of production types. It is worth mentioning that this alternative dataset has limitations. It predominantly assumed that the driving factors behind within-state and between-state shipments are the same, which may not hold. While this cross-comparison offers some qualitative support, the availability of real-world movement data in the future may strengthen the model validation, and result in different outcomes.

While this study focuses on Iowa, a swine densely populated state in US, the proposed approach can potentially estimate movement networks in other animal production systems or regions with proper adjustments. One of the main challenges lies in the data availability and granularity. Our model relies on input data including the swine movement flow distribution among different operation types, county-level swine sales and inventory data, individual farms’ operation type, inventory and location information. Depending on the data obtained, model parameters may differ from those used in this paper. In this paper, the percentages of movement flows among farms of different operation types were assumed based on previous literature [12] and may not generalize to other systems. For instance, in Table S1 (in supplemental material), we assume that 8.16% of the total movement are from nurseries to finishing farms and 19.71% of the total movement are from sow farms to nurseries. Furthermore, while we considered operation types during the network generation process (in algorithm 1), we did not use this information in the entropy optimization due to insufficient county-level sales data associated with operation types. Additional data collection efforts could possibly improve the estimation of movement probabilities considering operation types.

At larger geographic scales such as a country levels, the county-level sales and inventory data may not be readily available, and could be replaced by aggregated information (e.g., city-level statistics). In such cases, it may be more practical to generate movement networks, with each node representing a county or a few farms. However, this would lose the movement within a small region. It may be more suitable for high-density areas but not for geographically diverse areas. Nonetheless, the modeling framework presented in this paper can be adapted to other systems in future work.

## 5. Conclusion

Our results highlight the importance to incorporate livestock shipment data into modeling efforts as model outcomes are highly influenced by the network structure and properties. Network centrality measures such as out degree centrality was found to be very important in identifying critically important farms (i.e., potential super-spreaders). There is also a need to consider farm demographics and management practices such as operation type when generating synthetic networks, as later demonstrated, different operation types can greatly impact the network characteristics.

## Supporting information

S1 Supplemental material Swine movement patterns and generation. (DOCX)
