# Supplementary material for: Animal movement estimation and network-based epidemic modeling: Illustration for the swine industry in Iowa (US)
Source: PLoS One. 2025 Jun 18;20(6):e0326234. doi: 10.1371/journal.pone.0326234 (PMC12176235; doi:10.1371/journal.pone.0326234)
Supplement: S1 Supplemental material — (DOCX) [file pone.0326234.s001.docx]

Supplementary Information for

**A novel approach to estimate the animal movement networks: illustration for the swine industry in Iowa (US) and implications for disease prevention and control using a network-based model**

Qihui Yang^1*^, Beatriz Martínez-López^2^, Sifat Afroj Moon^3^, Jose Pablo Gomez-Vazquez^2^, Caterina Scoglio^1^

^1^ Department of Electrical and Computer Engineering, Kansas State University, Manhattan, KS, USA

^2^ Center for Animal Disease Modeling and Surveillance (CADMS), Department of Medicine & Epidemiology, School of Veterinary Medicine, University of California, Davis, CA, USA

^3^ Computing and Computational Sciences Directorate, Oak Ridge National Laboratory, Knoxville, TN, USA

* Corresponding author. [qihui@ksu.edu](mailto:qihui@ksu.edu)

**S1. Movement network estimation**

The *operation type mixing matrix* (Table S1) was obtained based on the number pigs transported from [1]. Values in the table correspond to fractions of movements from a row operation type to the corresponding column operation type, with the sum of all values in the table equaling 1. In other words, the table represents the pig movement fractions for any pair of operation types. For example, 0.0402 in Table 1 means that 4.02% of the total pig movements are from finishing farms to sow farms.

**Table S1. Operation type mixing matrix (fraction of movement from row type to column type)**

|  | Finishing farms | Gilt development unit (GDU) | Nursery | Sow farm | Wean-to-finish |
| --- | --- | --- | --- | --- | --- |
| Finishing farms | 0 | 0 | 0 | 0.0402 | 0 |
| GDU | 0 | 0.0569 | 0 | 0.1954 | 0 |
| Nursery | 0.0816 | 0 | 0 | 0 | 0 |
| Sow farm | 0 | 0.0358 | 0.1971 | 0 | 0.2754 |
| Wean-to-finish | 0.0563 | 0 | 0 | 0 | 0.0613 |

**S2. The individual-level network-based epidemic model**

To reduce the computational cost for the stochastic simulations, the pig inventory of each farm ranging from (0 – 999], (999 – 4999] and above 4,999 is scaled by factors of 1, 50, 500, respectively. After scaling, the total number of nodes in the network equals 466,316. The adjacency list is generated and imported to GEMF representing the network topology, where nodes represent pigs grouped by the 7,607 farms in Iowa. Links between nodes reflect both pig-to-pig contacts within a farm and pig movements between farms, affecting the disease spread. Links among pigs in each farm are generated based on the Erdős–Rényi model [2], and links among pigs from different farms are generated based on the estimated animal movement information. More specifically, let $C_{ij}$ represent the number of pigs moved daily from $i$ to farm $j$. Without the scaling factor, we have $\frac{dI_{j}}{dt}=S_{j}I_{j}D\beta+S_{j}I_{i}\frac{DC_{ij}}{N_{j}}\beta$, where $I_{j}$ and $S_{j}$ are the probabilities that an individual in farm *j* is in infected or susceptible, respectively; $I_{i}$ is the probability that an individual in farm $i$ is infected, and $N_{j}$ is the number of individuals in farm *j*. Considering scaling factors, the number of nodes in farms $i$ and $j$ are denoted as $N_{i}^{'}$ and $N_{j}^{'}$. Links between farms are weighted by $\frac{DC_{ij}}{N_{j}N_{i}^{'}}$, as illustrated in Figure 1 in the main text.

**Table S2. Swine movement patterns I**

Example of swine movement probabilities $m_{s1,s2,dist(c1,c2)}\times{10}^{3}$ from the maximum entropy approach

| Destination | | | | | | | | |
| --- | --- | --- | --- | --- | --- | --- | --- | --- |
|  | | Size 1 | Size 2 | Size 3 | Size 4 | Size 5 | Size 6 | Size 7 |
| Distance < 20 km | | | | | | | | |
| Source | Size 1 | 1.546 | 1.310 | 1.353 | 1.349 | 1.411 | 1.474 | 1.532 |
|  | Size 2 | 1.331 | 1.551 | 1.376 | 1.389 | 1.457 | 1.423 | 1.490 |
|  | Size 3 | 1.401 | 1.247 | 1.74 | 1.392 | 1.527 | 1.641 | 1.498 |
|  | Size 4 | 1.167 | 1.169 | 1.1950 | 2.373 | 1.865 | 1.831 | 2.077 |
|  | Size 5 | 0.746 | 0 | 0 | 1.792 | 6.617 | 4.460 | 5.110 |
|  | Size 6 | 0 | 0 | 0 | 1.081 | 7.192 | 13.080 | 8.220 |
|  | Size 7 | 0 | 0 | 0 | 0 | 0 | 0 | 16.290 |
| 20 km < Distance < 100 km | | | | | | | | |
| Source | Size 1 | 1.342 | 1.290 | 1.344 | 1.354 | 1.423 | 1.462 | 1.522 |
|  | Size 2 | 1.347 | 1.307 | 1.345 | 1.348 | 1.424 | 1.450 | 1.488 |
|  | Size 3 | 1.297 | 1.246 | 1.292 | 1.307 | 1.457 | 1.498 | 1.591 |
|  | Size 4 | 1.171 | 1.004 | 1.165 | 1.197 | 1.534 | 1.674 | 1.894 |
|  | Size 5 | 0 | 0 | 0 | 0.199 | 2.437 | 3.411 | 4.051 |
|  | Size 6 | 0 | 0 | 0 | 0 | 2.212 | 3.737 | 5.963 |
|  | Size 7 | 0 | 0 | 0 | 0 | 0 | 0 | 0 |
| 100 km < Distance < 200 km | | | | | | | | |
| Source | Size 1 | 1.336 | 1.282 | 1.330 | 1.344 | 1.418 | 1.457 | 1.515 |
|  | Size 2 | 1.345 | 1.299 | 1.340 | 1.359 | 1.412 | 1.442 | 1.489 |
|  | Size 3 | 1.327 | 1.218 | 1.285 | 1.327 | 1.425 | 1.498 | 1.601 |
|  | Size 4 | 1.199 | 1.005 | 1.171 | 1.227 | 1.521 | 1.637 | 1.899 |
|  | Size 5 | 0.235 | 0 | 0.032 | 0.686 | 1.766 | 2.515 | 4.124 |
|  | Size 6 | 0 | 0 | 0 | 0 | 0.483 | 2.295 | 5.834 |
|  | Size 7 | 0 | 0 | 0 | 0 | 0 | 0 | 0 |
| 200 km < Distance < 400 km | | | | | | | | |
| Source | Size 1 | 1.316 | 1.294 | 1.318 | 1.356 | 1.430 | 1.430 | 1.535 |
|  | Size 2 | 1.339 | 1.311 | 1.338 | 1.359 | 1.416 | 1.421 | 1.506 |
|  | Size 3 | 1.273 | 1.239 | 1.291 | 1.337 | 1.459 | 1.457 | 1.619 |
|  | Size 4 | 1.145 | 1.020 | 1.15 | 1.273 | 1.530 | 1.568 | 1.949 |
|  | Size 5 | 0.027 | 0 | 0.008 | 0.480 | 2.097 | 2.184 | 4.429 |
|  | Size 6 | 0 | 0 | 0 | 0 | 0.671 | 1.046 | 6.856 |
|  | Size 7 | 0 | 0 | 0 | 0 | 0 | 0 | 0 |
| Distance > 400 km | | | | | | | | |
| Source | Size 1 | 1.289 | 1.346 | 1.263 | 1.392 | 1.454 | 1.460 | 1.532 |
|  | Size 2 | 1.238 | 1.300 | 1.196 | 1.389 | 1.468 | 1.475 | 1.591 |
|  | Size 3 | 1.170 | 1.262 | 1.097 | 1.444 | 1.500 | 1.523 | 1.701 |
|  | Size 4 | 0.974 | 1.148 | 0.778 | 1.269 | 1.770 | 1.729 | 2.019 |
|  | Size 5 | 0 | 0 | 0 | 0 | 2.275 | 2.631 | 4.573 |
|  | Size 6 | 0 | 0 | 0 | 0 | 0.721 | 0.823 | 6.610 |
|  | Size 7 | 0 | 0 | 0 | 0 | 0 | 0 | 0 |

| **Algorithm 1**. Generation of animal movement networks | |
| --- | --- |
| **Input**: mixMatrix //Operation type mixing matrix.  totalPigMovement //Number of pigs moved per week.  farmList // Farm information with id, pig inventory, size group, county, operation type, latitude and longitude.  **A**  // Movement pattern.  counties, size_ groups, operation_types | |
| **Output**: Adjacency list of networks: Pig_Net, Farm_Net | |
| **Initialization:** Pig_Net = null  subpop_set = 3-dimensional null set // set of subpopulations  population = zeros(size(counties), size(size_ groups), size(operation_types))  distMatrix = zeros(size(counties), size(counties))  Farm_Net = zeros(nFarm,nFarm) // Weighted adjacency matrix | |
|  | **for each** *farm* in farmList //Generate subpopulations subpop_set (c,s,t) |
|  | Get the index vector of all pigs in *farm:* idx_pigs |
|  | Get *farm*’s indexes of county *c*, size group *s*, and operation type *t* |
|  | subpop_set(*c*, *s*, *t*).add(idx_pigs) //Add all pigs of this farm to corresponding subpopulation |
|  | population (*c*, s, *t*) = population (*c*, *s*, *t*) + size (idx_pigs) |
|  | **end for** |
|  |  |
|  | **for** i = 1 : size(operation_types) //Calculate the total number of pigs transported between each pair of operation types |
|  | **for** j = 1 : size(operation_types) |
|  | MovementFlowMatrix(i, j) **=** round**(**totalPigMovement*****mixMatrix(i, j)**)** // #pig to be moved between operation types i and j |
|  | **end for** |
|  | **end for** |
|  |  |
|  | **for** i = 1 : size(counties) |
|  | **for** j = 1 : size(counties) |
|  | distMatrix(i, j) **=** get_distance(i, j) // distMatrix records the distance category between each pair of counties |
|  | **end for** |
|  | **end for** |
|  |  |
|  | nLink **=** 1 // Build pig network |
|  | **for** *t1* **=** 1 : size(operation_types) |
|  | **for** *t2* **=** 1 : size(operation_types) |
|  | num_Pig2Move **=** MovementFlowMatrix**(**t1, t2**)** |
|  | **if** **(**num_Pig2Move **==** 0**)** **continue** **end** |
|  | **for** i = 1 : num_Pig2Move |
|  | flag **=** **True** |
|  | **while** **(**flag**)** |
|  | **[***s1*, *s2*, *dist***] =** rand_based_on_probability**(A)** //randomly select a source size group, a destination size group and distance |
|  | position **=** find**(**distMatrix**==***dist***)** // Find the list of county pairs with distance equaling dist |
|  | randomCountyPair **=** position**(**randperm**(**size**(**position**)))** //Shuffle the positions |
|  | **for** j = 1: size(randomCountyPair) // Traverse all pair in case population (*c1*, *s1*, *t1*) or population (*c2*, *s2*, *t2*) equal to 0 |
|  | *c1*= randomCountyPair(j, 1); *c2* = randomCountyPair(j, 2) |
|  | **if** ( (population(*c1*, *s1*, *t1*)>0) & (population(*c2, s2, t2*)>0) ) // Ensure the selected subpopulations have pigs |
|  | idx_pig1 = rand_select_one(subpop_set (*c1*, *s1*, *t1*)) // Randomly select one pig from subpopulation (*c1*,s1,t1) |
|  | idx_pig2 = rand_select_one(subpop_set (*c2*, *s2*, *t2*)) |
|  | Pig_Net**(**nLink, 1**) =** idx_pig1; Pig_Net(nLink, 2) = idx_pig2 //Add a link between selected pigs |
|  | farm_from = getFarmIndex(idx_pig1) // Get farm indexes of the selected pigs |
|  | farm_to = getFarmIndex(idx_pig2) |
|  | Farm_Net(farm_from, farm_to)++ |
|  | subpop_set(*c1, s1, t1*).remove(idx_pig1) // Remove idx_pig1 from subpopulation subpop_set(*c1, s1, t1*) |
|  | population(*c1*, *s1*, *t1*) -- // Decrement population (*c1, s1, t1*) by 1. |
|  | nLink **++** // Number of links |
|  | flag **= False** |
|  | **break** |
|  | **end** **if** |
|  | **end for** |
|  | **end while** |
|  | **end for** |
|  | **end for** |
|  | **end for** |

**S3. Additional results based on swine movement patterns II**

Based on the synthetic networks provided by Sellman et al. [3], which are generated based on interstate shipment data, we extract the intra-state shipments for Iowa. We then categorize the travel distance of each movement record to the five categories, based on the county centroids of the origin and destination. Second, we count the number pigs moved for each pair of size group, within a distance category. We can then divide that number of pigs by the total movement, as shown in Table S3. Here, we consider 9 size groups: 1 – 24 pigs, 25 – 49 pigs, 50 – 99 pigs, 100 – 199 pigs, 200 – 499 pigs, 500 – 999 pigs, and 1000 – 4999, 5000 – 9999 pigs, and more than 10000 pigs.

**Table S3. Swine movement patterns II**

Example of swine movement probabilities $m_{s1,s2,dist(c1,c2)}\times{10}^{3}$ **from Sellman et al.**

| Destination | | | | | | | | | | |
| --- | --- | --- | --- | --- | --- | --- | --- | --- | --- | --- |
|  | | Size 1 | Size 2 | Size 3 | Size 4 | Size 5 | Size 6 | Size 7 | Size 8 | Size 9 |
| Distance < 20 km | | | | | | | | |  |  |
| Source | Size 1 | 0.3985 | 0.084 | 0.0973 | 0.1541 | 0.4119 | 0.482 | 2.0823 | 1.845 | 1.4607 |
|  | Size 2 | 0.0767 | 0.0362 | 0.0311 | 0.0272 | 0.0815 | 0.1041 | 0.3903 | 0.3282 | 0.2017 |
|  | Size 3 | 0.091 | 0.0209 | 0.0451 | 0.0404 | 0.117 | 0.1386 | 0.778 | 0.5451 | 0.3454 |
|  | Size 4 | 0.1148 | 0.0274 | 0.0346 | 0.0595 | 0.1741 | 0.2175 | 0.845 | 0.7611 | 0.5154 |
|  | Size 5 | 0.3385 | 0.0843 | 0.1893 | 0.1964 | 0.6498 | 0.824 | 3.4038 | 3.2106 | 2.1694 |
|  | Size 6 | 0.53 | 0.0861 | 0.1697 | 0.384 | 0.8424 | 1.2615 | 5.124 | 3.8067 | 1.6209 |
|  | Size 7 | 1.6948 | 0.304 | 0.4779 | 0.6719 | 2.9542 | 4.3445 | 21.1585 | 14.6558 | 8.1309 |
|  | Size 8 | 1.1995 | 0.2268 | 0.3561 | 0.5998 | 2.7683 | 3.3307 | 12.9168 | 15.8631 | 6.8634 |
|  | Size 9 | 1.1457 | 0.1285 | 0.4953 | 0.531 | 1.2767 | 1.5658 | 4.6956 | 9.6127 | 13.1139 |
| 20 km < Distance < 100 km | | | | | | | | |  |  |
| Source | Size 1 | 1.3789 | 0.3397 | 0.4178 | 0.5534 | 1.9761 | 2.4894 | 10.4359 | 8.5874 | 3.9871 |
|  | Size 2 | 0.3223 | 0.0646 | 0.0934 | 0.1213 | 0.3996 | 0.5188 | 2.2583 | 1.7268 | 0.744 |
|  | Size 3 | 0.3792 | 0.0867 | 0.1152 | 0.1677 | 0.6015 | 0.7445 | 3.1091 | 2.8047 | 1.4062 |
|  | Size 4 | 0.4889 | 0.1151 | 0.1577 | 0.2016 | 0.7353 | 0.9576 | 4.0775 | 3.435 | 1.5107 |
|  | Size 5 | 1.3931 | 0.3339 | 0.4794 | 0.6279 | 2.3527 | 3.1014 | 13.9264 | 10.8762 | 4.7839 |
|  | Size 6 | 1.8392 | 0.4051 | 0.5437 | 0.772 | 3.0766 | 4.0912 | 18.9614 | 14.1554 | 6.0549 |
|  | Size 7 | 6.4667 | 1.5595 | 2.0857 | 2.8967 | 12.0862 | 16.425 | 75.4986 | 54.9625 | 23.3816 |
|  | Size 8 | 4.4403 | 1.0483 | 1.4952 | 2.0387 | 8.4488 | 10.9733 | 49.19 | 38.9232 | 17.5521 |
|  | Size 9 | 1.6084 | 0.3054 | 0.5812 | 0.6836 | 2.7193 | 3.1656 | 14.7268 | 13.953 | 9.6023 |
| 100 km < Distance < 200 km | | | | | | | | |  |  |
| Source | Size 1 | 0.6219 | 0.1453 | 0.1936 | 0.2672 | 0.9816 | 1.2217 | 5.188 | 4.2796 | 2.264 |
|  | Size 2 | 0.1453 | 0.037 | 0.0439 | 0.0536 | 0.223 | 0.2832 | 1.1429 | 0.9907 | 0.5548 |
|  | Size 3 | 0.1707 | 0.041 | 0.045 | 0.0709 | 0.2712 | 0.3658 | 1.5937 | 1.2279 | 0.5532 |
|  | Size 4 | 0.2217 | 0.0489 | 0.0679 | 0.0908 | 0.3487 | 0.4617 | 1.9533 | 1.5891 | 0.8096 |
|  | Size 5 | 0.6344 | 0.1455 | 0.206 | 0.2957 | 1.074 | 1.3303 | 6.0956 | 4.773 | 2.3749 |
|  | Size 6 | 0.834 | 0.1941 | 0.2641 | 0.3591 | 1.3525 | 1.6815 | 7.4054 | 6.1217 | 3.1287 |
|  | Size 7 | 2.9388 | 0.718 | 0.9598 | 1.36 | 4.8942 | 6.2088 | 27.1657 | 22.1916 | 11.3001 |
|  | Size 8 | 2.1093 | 0.4793 | 0.6843 | 0.9507 | 3.5048 | 4.6139 | 20.6641 | 15.7672 | 7.9555 |
|  | Size 9 | 0.7529 | 0.196 | 0.2506 | 0.3648 | 1.3438 | 1.8708 | 8.3503 | 6.5124 | 2.3272 |
| 200 km < Distance < 400 km | | | | | | | | |  |  |
| Source | Size 1 | 0.2316 | 0.053 | 0.0825 | 0.1078 | 0.3988 | 0.5408 | 2.6325 | 1.9099 | 0.6604 |
|  | Size 2 | 0.0523 | 0.0142 | 0.0187 | 0.0271 | 0.0972 | 0.1384 | 0.6386 | 0.4372 | 0.1738 |
|  | Size 3 | 0.0696 | 0.0149 | 0.0217 | 0.0302 | 0.1069 | 0.1392 | 0.7158 | 0.5091 | 0.1946 |
|  | Size 4 | 0.0812 | 0.0197 | 0.0235 | 0.0362 | 0.1421 | 0.196 | 0.8014 | 0.6216 | 0.2321 |
|  | Size 5 | 0.2287 | 0.0588 | 0.0777 | 0.096 | 0.3981 | 0.5124 | 2.3884 | 1.7476 | 0.6335 |
|  | Size 6 | 0.2974 | 0.0647 | 0.0854 | 0.1268 | 0.4691 | 0.666 | 2.9454 | 2.2575 | 0.825 |
|  | Size 7 | 1.0968 | 0.2791 | 0.3515 | 0.4677 | 1.7523 | 2.3963 | 11.1043 | 8.0864 | 3.1304 |
|  | Size 8 | 0.7524 | 0.1883 | 0.2271 | 0.3365 | 1.2552 | 1.6755 | 7.635 | 5.6954 | 2.0035 |
|  | Size 9 | 0.2613 | 0.0595 | 0.0763 | 0.1051 | 0.4346 | 0.6263 | 3.0308 | 2.0751 | 0.8517 |
| Distance > 400 km | | | | | | | | |  |  |
| Source | Size 1 | 0.0054 | 0.0015 | 0.003 | 0.0025 | 0.0122 | 0.0265 | 0.154 | 0.0687 | 0.0029 |
|  | Size 2 | 0.0008 | 0.0003 | 0.0001 | 0.0008 | 0.0035 | 0.0064 | 0.0477 | 0.0165 | 0.0002 |
|  | Size 3 | 0.0018 | 0.001 | 0.0012 | 0.0007 | 0.0053 | 0.0084 | 0.0513 | 0.0243 | 0.0004 |
|  | Size 4 | 0.0011 | 0.0002 | 0.001 | 0.0006 | 0.0058 | 0.0107 | 0.0558 | 0.0257 | 0.0001 |
|  | Size 5 | 0.0047 | 0.0015 | 0.0023 | 0.0038 | 0.0169 | 0.0303 | 0.1529 | 0.0734 | 0.008 |
|  | Size 6 | 0.0079 | 0.0022 | 0.0024 | 0.0041 | 0.021 | 0.0357 | 0.2113 | 0.1008 | 0.0081 |
|  | Size 7 | 0.0318 | 0.0103 | 0.012 | 0.0163 | 0.078 | 0.1357 | 0.6501 | 0.3007 | 0.0516 |
|  | Size 8 | 0.0172 | 0.0071 | 0.0056 | 0.0085 | 0.0428 | 0.0557 | 0.3793 | 0.1966 | 0.0208 |
|  | Size 9 | 0.0055 | 0.0016 | 0.0005 | 0.0013 | 0.0101 | 0.0172 | 0.1389 | 0.0537 | 0 |

We generate weekly farm networks following the approach described in the main text with swine movement pattern II (in Table S3) and perform network analyses. The results are shown in Table S4 and Figures S1 and S2.

**(a)**

**
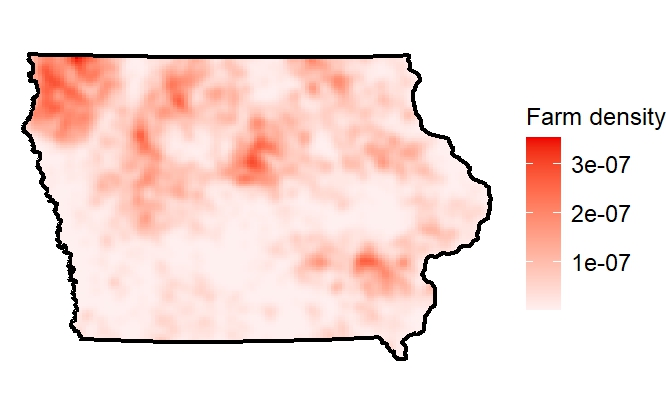
**

**(b)**


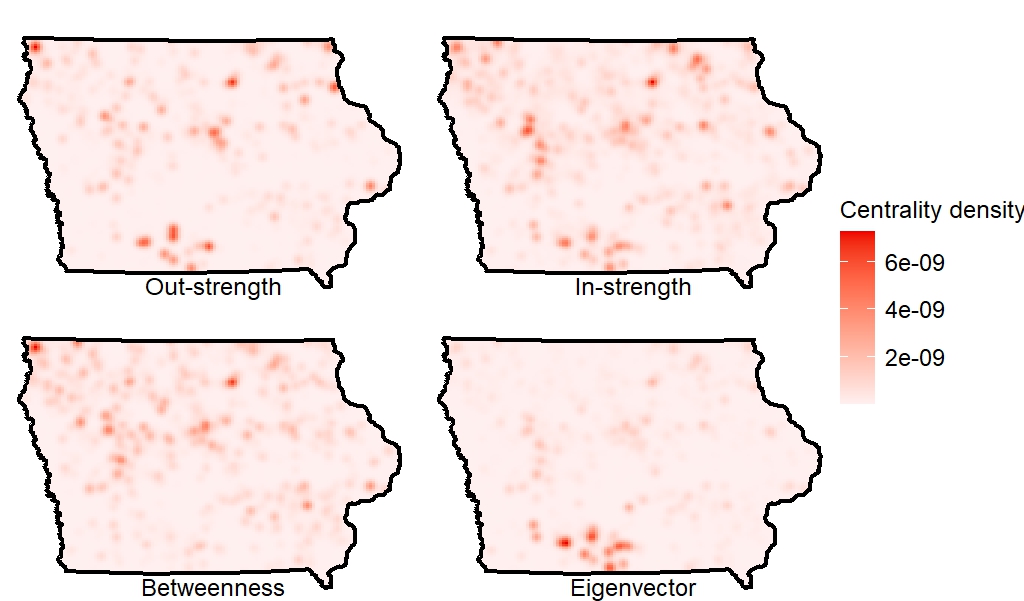


Figure S1. Distributions of (a) farm density and (b) network centrality measures: out-strength, in-strength, betweenness, and eigenvector centrality of the swine movements at the level of premises. On the map, the raster with the red color scale was produced using the median value of the network metrics from the 200 synthetic networks. Here, swine movement networks were generated using the movement patterns described in Table S3.

**(a) (b)**


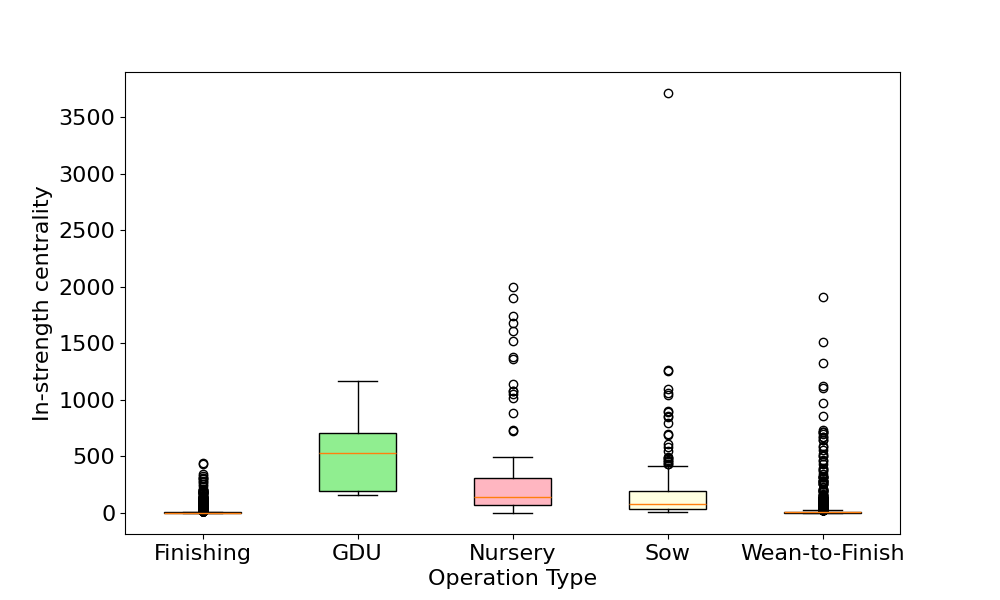

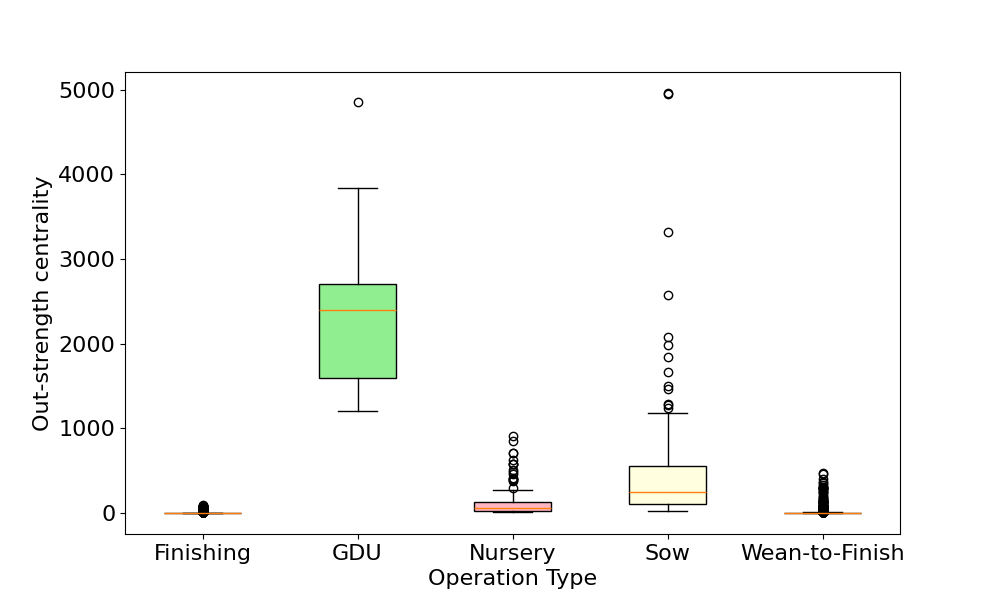


**(c) (d)**


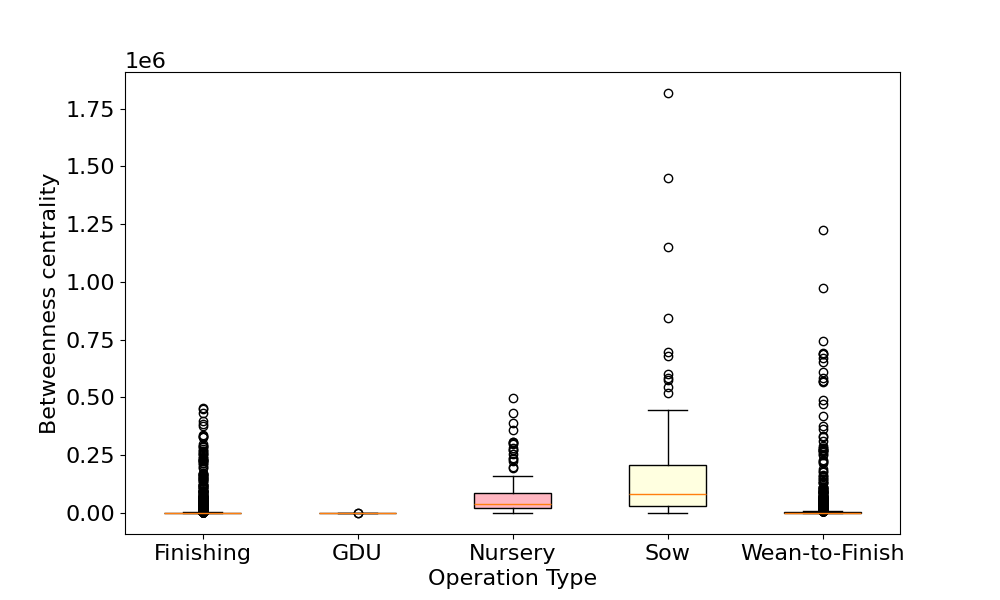

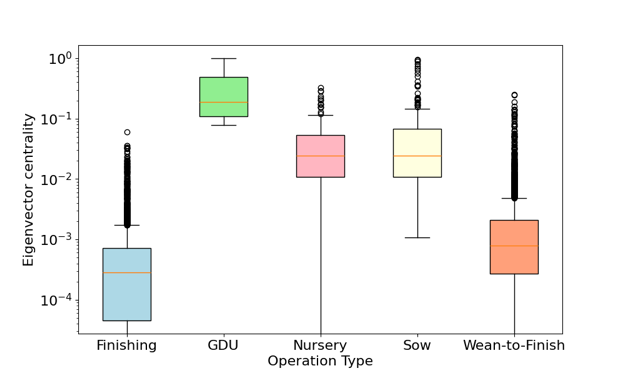


Figure S2. Plots of centrality measures for each production type. The plots show the distributions of (a) in-strength, (b) out-strength, (c) betweenness, and (d) eigenvector centrality. The figure is based on network metric values calculated from the 200 synthetic swine movement networks. These networks were generated using the movement patterns described in Table S3.

**Table S4. General network properties of the generated weekly networks**

| **Network measure** | **Average (minimum, maximum)** |
| --- | --- |
| Number of edges | 87458 (87002, 87494) |
| Number of nodes | 7248 (7205, 7284) |
| Density ($\times{10}^{3}$) | 1.66 (1.65, 1.69) |
| Diameter | 8.71 (8.00, 10.00) |
| Average shortest path | 3.57 (3.55, 3.59) |
| Transitivity (undirected) | 0.048 (0.047, 0.049) |
| Number of weakly connected components | 1 (1, 5) |
| Number of strongly connected components | 1962 (1880, 2024) |
| Size of the largest strongly connected component (%) | 69.27 (68.28, 70.40) |
| Size of the largest weakly connected component (%) | 95.27 (94.72, 95.75) |
| Mean betweenness | 14534.88 (14207.04,14836.01 ) |
| Mean eigenvector ($\times{10}^{3}$) | 6.56 (5.93, 7.07) |
| Average in-degree | 12.07 (11.99, 12.14) |
| Average out-degree | 12.07 (11.99, 12.14) |

**References**

[1] Lee K, Polson D, Lowe E, Main R, Holtkamp D, Martínez-López B. Unraveling the contact patterns and network structure of pig shipments in the United States and its association with porcine reproductive and respiratory syndrome virus (PRRSV) outbreaks. Prev Vet Med. 2017 Mar 1; 138:113–23. doi: 10.1016/j.prevetmed.2017.02.001.

[2] Erdős P, Rényi A. On random graphs I. Publ Math. 1958;6:290–7.

[3] Sellman S, Beck-Johnson LM, Hallman C, Miller RS, Owers Bonner KA, Portacci K, et al. Modeling nation-wide U.S. swine movement networks at the resolution of the individual premises. Epidemics. 2022 Dec 1; 41:100636. doi: 10.1016/j.epidem.2022.100636.
